# Growth factor betacellulin can stimulate human granulosa cell functions and promote gonadotropin actions

**DOI:** 10.1007/s11033-026-12091-4

**Published:** 2026-06-04

**Authors:** Barbora Loncová, Zuzana Fabová, Miloš Mlynček, Alexander V. Sirotkin

**Affiliations:** Constantine the Philosopher University in Nitra, Tr. A. Hlinku 1, Nitra, 949 74 Slovakia

**Keywords:** Betacellulin, FSH, LH, Ovary, Human, Steroid hormones, Proliferation, Apoptosis

## Abstract

**Background:**

Betacellulin (BTC), an EGF-family growth factor expressed in granulosa cells, is implicated in ovarian regulation; however, its interactions with gonadotropins in humans remain unclear.

**Objective:**

To determine how BTC alone or combined with follicle-stimulating hormone (FSH) or luteinizing hormone (LH) affects human granulosa cell viability, proliferation, apoptosis, and progesterone secretion.

**Methods:**

Human granulosa cells were treated with BTC (1–100 ng/ml) ± FSH or LH (100 ng/ml). Viability was measured by Trypan blue exclusion; proliferation and apoptosis by immunocytochemistry; and progesterone by ELISA.

**Results:**

BTC dose-dependently increased viability, proliferation (cyclin B1, PCNA), and progesterone secretion, while reducing apoptosis (bax, caspase-3). Both FSH and LH enhanced all BTC effects, further increasing proliferation, viability, progesterone output, and anti-apoptotic activity.

**Conclusion:**

BTC may regulate human granulosa cell function, acting in concert with gonadotropins to modulate viability, proliferation, apoptosis, and steroidogenesis, highlighting its role as a paracrine mediator in ovarian physiology.

## Introduction

Successful reproduction depends on the integration of multiple factors that regulate ovarian processes and cell functions through endocrine and paracrine signaling. Gonadotropins are key regulators of ovarian function, regulating granulosa and theca cell proliferation, survival, and steroidogenesis, and supporting follicular development and oocyte maturation. In addition, they suppress apoptosis and promote ovulation and luteinization, thereby ensuring normal reproductive capacity [1, 2, 3]. In addition to gonadotropins, growth factors play important roles in regulating ovarian cell function. One such growth factor is betacellulin (BTC), a ligand of the epidermal growth factor receptor, which has been detected in ovarian granulosa cells of multiple species, including humans [4, 5, 6], suggesting a critical role in ovarian function.

Previous studies in animals have shown that BTC has multiple biological functions in the ovary: it can influence proliferation and apoptosis in cultured ovarian cells [7, 8], affect steroid hormone production depending on the species, stimulating progesterone and estradiol production in pigs [8], while suppressing steroid hormone release in cats [7]. Moreover, BTC may promote prostaglandin production in humans [4, 9]. These findings indicate the multifunctional role of BTC in the modulation of ovarian cell activity. Importantly, there is evidence for functional interactions between BTC and gonadotropins. Gonadotropins can induce or modulate EGF-like ligand expression, including BTC, in mice [10, 11], cattle [12], pigs [13], and human [5] granulosa cells. BTC has been associated with ovulation-related processes downstream of LH signaling in rodents [9, 14], promoting cumulus-oocyte complex maturation and developmental competence in vitro in pigs [12] and rodents [14], and is a potential paracrine mediator of gonadotropin action within the ovulatory follicle in mammals [4, 8].

Although BTC expression in human granulosa cells is established, its interactions with gonadotropins (FSH and LH) and their effects on key functional processes involved in granulosa cell viability, proliferation, apoptosis, and progesterone secretion remain poorly defined.

Understanding these interactions is therefore crucial to determining whether BTC is a central regulator of ovarian function and a central integrator of gonadotropin signaling in humans.

Thus, this study aimed to clarify the role of BTC and its interactions with gonadotropins in the regulation of these critical functions in human ovarian granulosa cells, with particular emphasis on how gonadotropins modulate BTC action.

## Materials and methods

### Preparation, culture, and processing of granulosa cells

Sixteen ovaries with normal morphology were obtained from women aged 36–42 years during the follicular phase, undergoing ovariectomy for non-metastatic cervical cancer. The donors had not received hormonal treatment before surgery. Informed consent was secured in accordance with EU and Slovak ethical regulations and approved by the Ethics Committee of the University Hospital of Nitra. Granulosa cells were aspirated from visually healthy, non-atretic (defined by the absence of weak vascularization, thin follicular walls, or pale follicular fluid) follicles (2–6 mm), isolated by centrifugation (1500 rpm, 10 min), and washed in a 1:1 DMEM/Ham’s F-12 medium (BioWhittaker TM, Verviers, Belgium) supplemented with 10% fetal bovine serum (South America origin, Bio-West Inc., Logan, UT, USA) and 1% antibiotic–antimycotic solution (Sigma-Aldrich, St. Louis, MO, USA). Cell concentration and viability were evaluated by Trypan blue exclusion. Cells were cultured in 24-well plates (1 ml per well, Nunc, A/S, Roskilde, Denmark) and chamber slides (100 µl per well, Nunc Inc., International, Naperville, IL) at 10⁴–10⁵ cells/ml. Cells were cultured at 37 °C in a humidified incubator with 5% CO₂. Humidity (~ 95%) was maintained by a water reservoir within the incubator. Following a 48-h preculture, media were replaced, and cells were treated with BTC (1, 10, 100 ng/ml) alone or in combination with FSH (100 ng/ml) or LH (100 ng/ml) (all from Sigma-Aldrich). The lowest concentration (1 ng/ml) corresponds to physiologically relevant BTC levels, whereas 10 and 100 ng/ml represent supra-physiological concentrations, similar to those used in previous in vitro studies [6, 15]. FSH and LH were applied at concentrations commonly used in in vitro studies [16], which exceed physiological serum levels. Untreated cells served as controls. After 48 h, the media were stored at − 20 °C until ELISA analysis, and cell monolayers were fixed with 4% paraformaldehyde for immunocytochemical analysis.

### Trypan blue exclusion test

The Trypan blue exclusion assay was used to assess the effects of BTC, FSH, LH, and their combinations on granulosa cell viability, following Perry et al. [17] and Strober [18]. After incubation, the culture medium was removed and the cell monolayers were stained with 0.4% Trypan blue (15 min), rinsed twice with physiological solution, and fixed in 4% paraformaldehyde for 30 min. After an additional wash, cells were examined under a microscope at 400× magnification. Nonviable, dye-positive cells were counted, and viability was expressed as the proportion of dead cells relative to the total cells.

### Immunocytochemical analysis

Proliferation markers (PCNA, cyclin B1) and apoptosis markers (bax, caspase 3) in granulosa cells cultured on chamber slides were assessed by immunocytochemistry [19, 20]. Following washing and fixation of the cells, cells were blocked with 1% goat serum (in PBS) for 1 h and subsequently incubated for 1 h with mouse monoclonal antibodies against PCNA (Santa Cruz Biotechnology, sc-25280), cyclin B1 (Santa Cruz Biotechnology, sc-245), bax (Santa Cruz Biotechnology, sc-23959), or caspase 3 (Santa Cruz Biotechnology, sc-7272) (all diluted 1:500). The primary antibodies used in this study have been validated for immunocytochemistry by the manufacturer and in previous studies. Visualization was achieved using an HRP-conjugated goat anti-mouse IgG secondary antibody (Santa Cruz Biotechnology, sc-516102) (1:100 dilution, 1 h) and DAB substrate. After washing with PBS, cells were mounted with Vectashield. Negative controls were processed without the primary antibody and showed no specific staining. Immunopositive cells, identified by brown DAB staining using light microscopy, were quantified by counting 1000 cells per chamber (3000 per group). Cell counting was performed by two blinded observers using predefined and consistent criteria to minimize subjective bias. The proportion of antigen-positive cells was calculated.

### Enzyme-linked immunosorbent assay (ELISA)

Progesterone levels were quantified in 25 µl aliquots using the corresponding ELISA kit (LDN Immunoassays and Services, Nordhorn, Germany). All measurements were performed in duplicate. Secretion rates were expressed per 10⁶ viable cells per day. The measured concentration of progesterone in the culture media fell within the linear range of the respective ELISA kit (0.140–40 ng/ml).

Antiserum against progesterone cross-reacted by ≤ 1.1% with 11-desoxycorticosterone, by ≤ 0.35% with pregnenolone, by ≤ 0.30% with 17α -hydroxyprogesterone, by ≤ 0.20% with corticosterone, by ˂ 0.10% with estriol, 17β-E2, T, cortisone, and 11-desoxycortisol, by ˂ 0.02% with dehydroepiandrosterone sulfate and cortisol. Intra- and inter-assay coefficients of variation did not exceed 5.40 and 5.59%, respectively. The sensitivity of the assay was 0.045 ng/ml.

### Statistical analysis

The presented results summarize three independent experiments (*n* = 3) performed on separate days using different groups of human granulosa cells, each obtained from a minimum of five ovaries. Each experiment represents an independent biological replicate derived from different donors. The effects of BTC treatment (three concentrations) and gonadotropin stimulation (presence or absence of FSH or LH) were evaluated using two-way ANOVA in a factorial design, allowing assessment of the main effects of each factor as well as their interaction. When significant effects were detected, Tukey’s post-hoc test was applied for multiple comparisons (SigmaPlot 11.0). Statistical significance was defined as *P* < 0.05. Data are presented as means ± SEM. Comparisons were made within each experiment relative to its corresponding control group. Due to limited sample size and experimental design, inter-donor variability was not included as a factor in the statistical model.

## Results

### The effect of BTC on ovarian cell functions

**Fig. 1:**
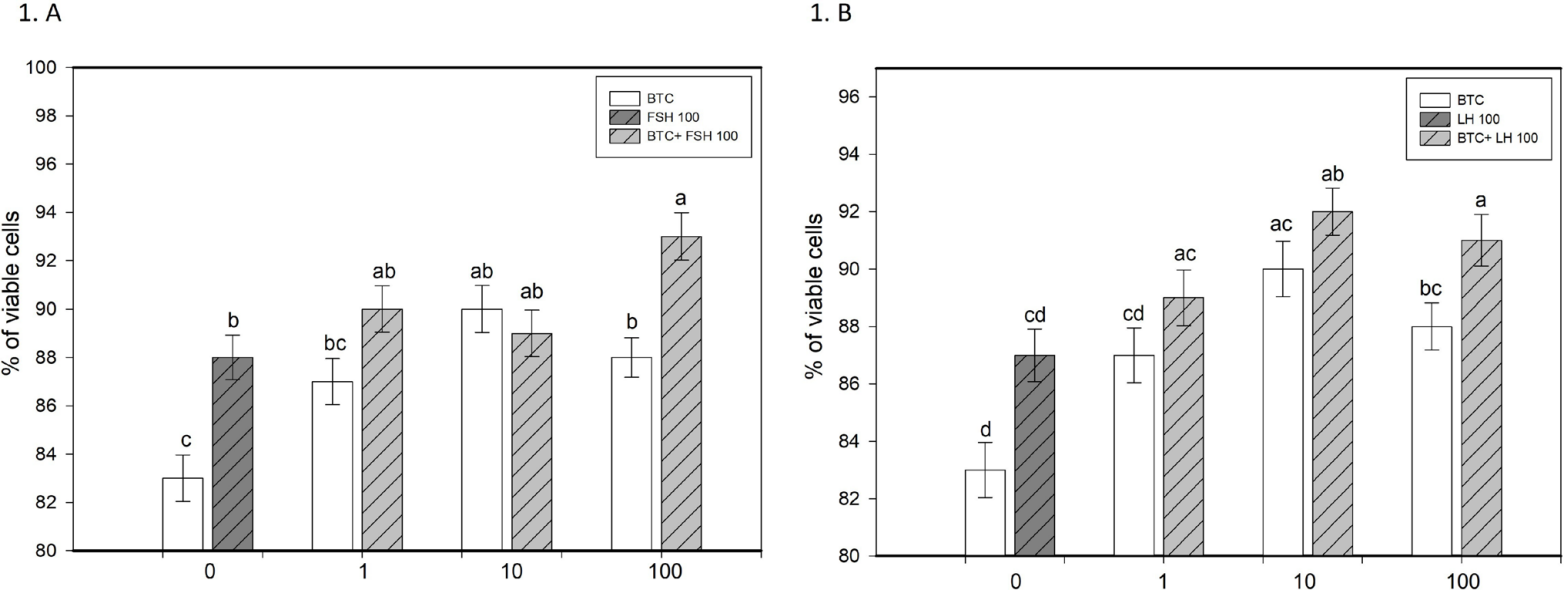
Effects of betacellulin (BTC; 0, 10, or 100 ng/ml), FSH (100 ng/ml), LH (100 ng/ml), and their combinations on human granulosa cell viability. (**A**) Percentage of viable cells after treatment with increasing concentrations of BTC (0, 1, 10, 100) in the presence of FSH (100) or BTC + FSH (100). (**B**) Percentage of viable cells after treatment with increasing concentrations of BTC (0, 1, 10, 100) in the presence of LH (100) or BTC + LH (100). Data are presented as mean ± SD. Different letters above bars indicate statistically significant differences (*p* < 0.05)

**Fig. 2:**
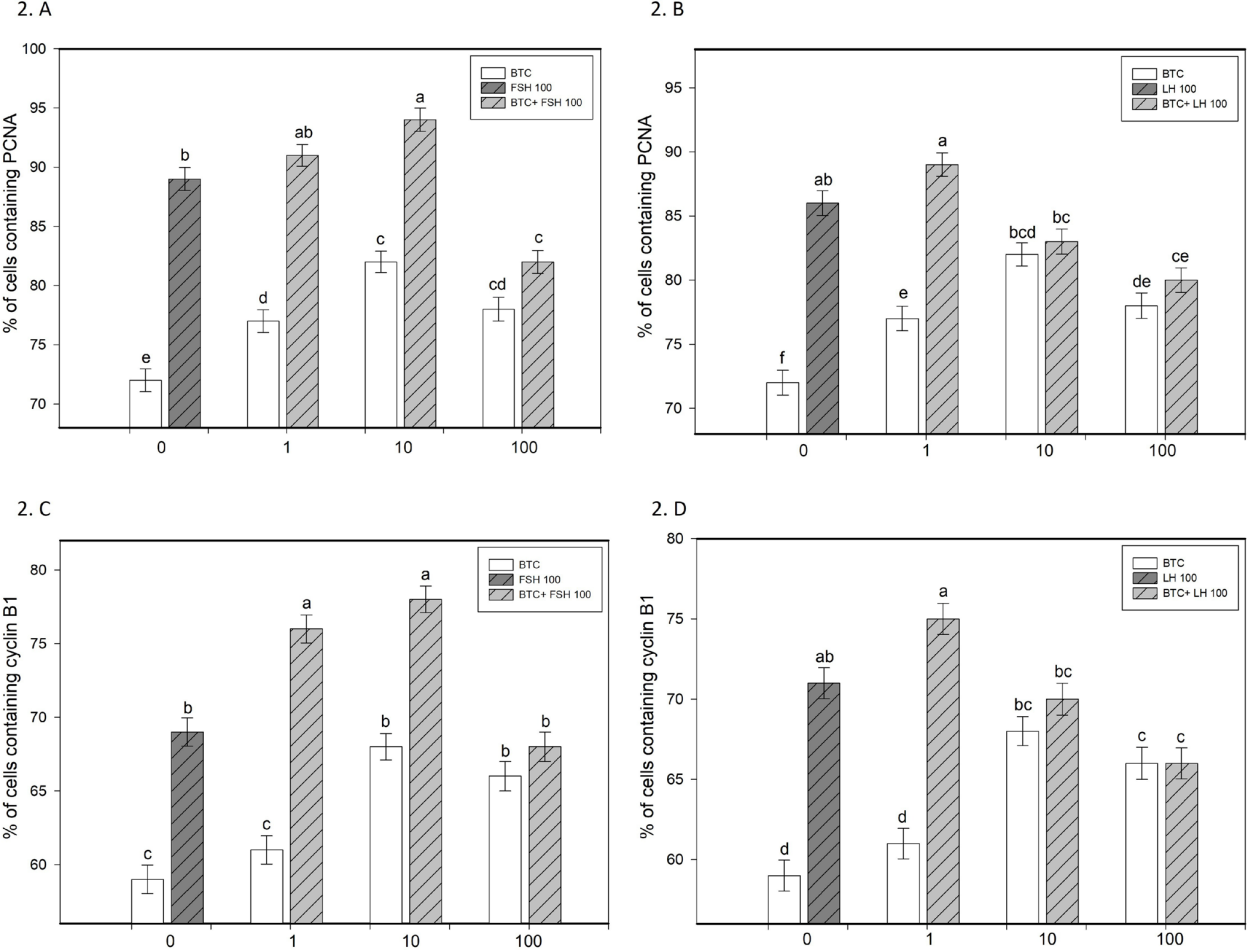
Effects of FSH and LH, alone or in combination with BTC, on proliferation markers PCNA and cyclin B1. (**A**, **B**) Percentage of cells containing PCNA following treatment with BTC (0, 1, 10, 100) in the presence of FSH (100) (**A**) or LH (100) (**B**). (**C**, **D**) Percentage of cells containing cyclin B1 following treatment with BTC (0, 1, 10, 100) in the presence of FSH (100) (**C**) or LH (100) (**D**). Data are presented as mean ± SD. Different letters above bars indicate statistically significant differences among groups (*p* < 0.05)

**Fig. 3:**
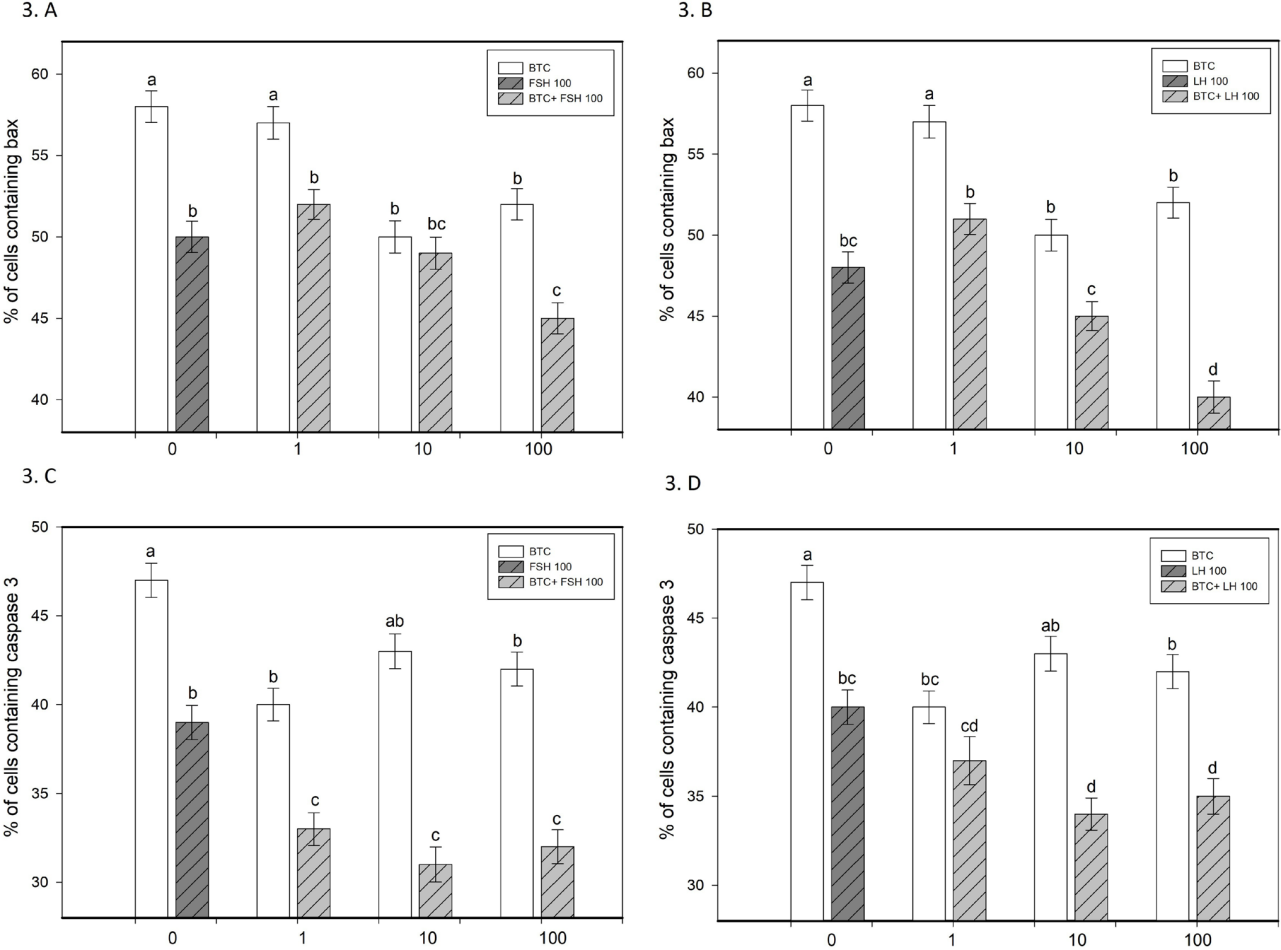
Effects of FSH and LH, alone or in combination with BTC, on apoptotic markers (Bax and caspase 3). (**A**, **B**) Percentage of cells containing Bax following treatment with BTC (0, 1, 10, 100) in the presence of FSH (100) (**A**) or LH (100) (**B**). (**C**, **D**) Percentage of cells containing caspase 3 following treatment with BTC (0, 1, 10, 100) in the presence of FSH (100) (**C**) or LH (100) (**D**). Data are presented as mean ± SD. Different letters above bars indicate statistically significant differences among groups (*p* < 0.05)

**Fig. 4:**
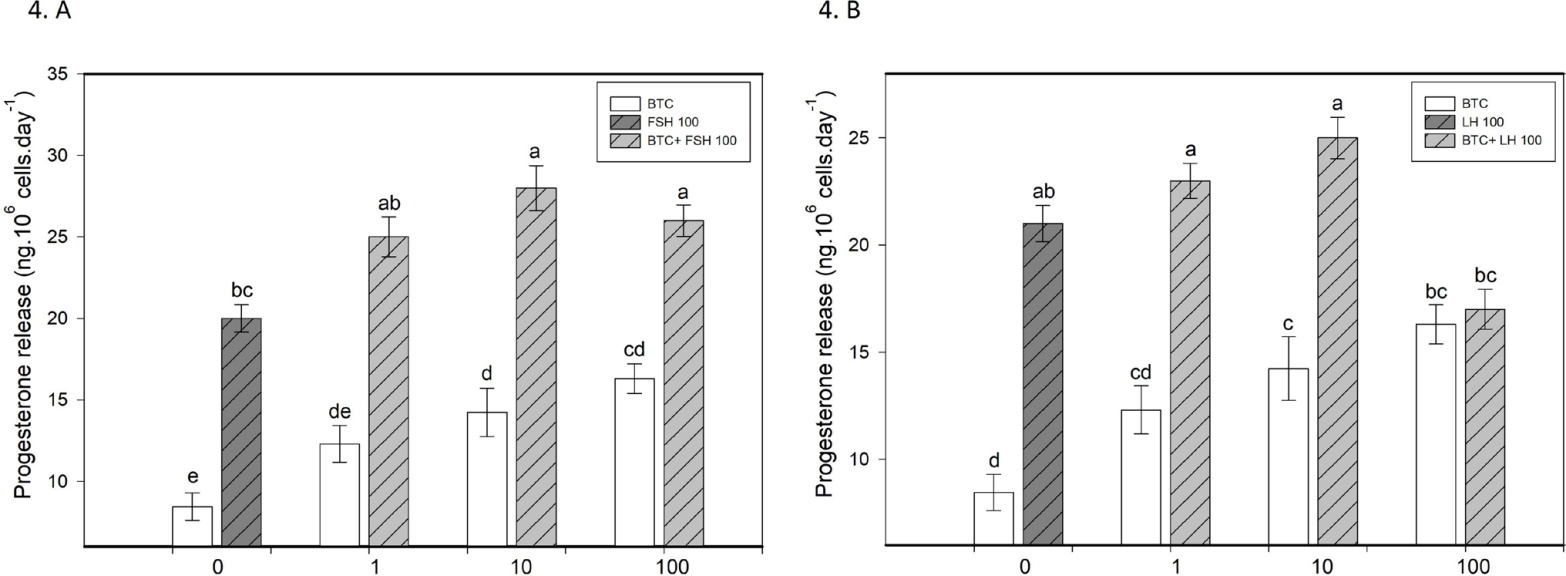
Effects of FSH and LH, alone or in combination with BTC, on progesterone release. (**A**,** B**) Progesterone secretion (ng·10⁶ cells·day⁻¹) following treatment with BTC (0, 1, 10, 100) in the presence of FSH (100) (**A**) or LH (100) (**B**). Data are presented as mean ± SD. Different letters above bars indicate statistically significant differences among groups (*p* < 0.05)

BTC increased granulosa cell viability and progesterone secretion, particularly at 10 and 100 ng/ml (*p* < 0.05; Figs. 1A and B and 4A and B). It also promoted proliferation, as indicated by increased proportions of PCNA-positive cells at all tested concentrations and cyclin B1-positive cells at 10 and 100 ng/ml (*p* < 0.05; Fig. 2A–D). In parallel, BTC reduced apoptotic markers (bax and caspase-3), with more pronounced effects at 10 and 100 ng/ml (*p* < 0.05; Fig. 3A–D).

### The effect of FSH and LH on ovarian cell functions

FSH and LH (100 ng/ml) increased granulosa cell viability, promoted proliferation (PCNA, cyclin B1), reduced apoptosis (bax and caspase-3), and stimulated progesterone secretion compared to controls (all *p* < 0.05; Figs. 1, 2, 3 and 4).

### The effect of BTC in combination with FSH on ovarian cell functions

The combined treatment of BTC and FSH showed concentration-dependent effects on granulosa cell function. BTC enhanced the stimulatory effect of FSH on viability at 100 ng/ml and increased FSH-induced proliferation, particularly at 10 ng/ml, as indicated by PCNA and at 1 and 10 ng/ml, as indicated by cyclin B1 accumulation (*p* < 0.05; Figs. 1A and 2A and C). In addition, BTC supported the inhibitory effect of FSH on apoptotic markers, especially at 100 ng/ml for bax and across all tested concentrations for caspase-3 (*p* < 0.05; Fig. 3A, C). BTC also potentiated FSH-stimulated progesterone secretion at 10 and 100 ng/ml (*p* < 0.05; Fig. 4A).

Conversely, FSH supported BTC-induced effects on viability (at 1 and 100 ng/ml), proliferation (at 1 and 10 ng/ml), progesterone release (at all concentrations), and apoptosis (bax at 1 and 100 ng/ml; caspase-3 at all concentrations), compared with the corresponding BTC concentration alone (all *p* < 0.05).

### The effect of BTC in combination with LH on ovarian cell functions

The effects of BTC in combination with LH were concentration-dependent. BTC enhanced LH-induced increases in viability at 10 and 100 ng/ml and promoted proliferation (PCNA, cyclin B1) at 100 ng/ml (all *p* < 0.05; Figs. 1B and 2B and D). The anti-apoptotic effects of LH were further supported by BTC, particularly at higher concentrations (bax at 100 ng/ml; caspase-3 at 1–100 ng/ml) (*p* < 0.05; Fig. 3B, D). However, BTC did not significantly modify LH-stimulated progesterone secretion (Fig. 4B).

Conversely, LH supported BTC-induced effects on viability at 100 ng/ml, proliferation (PCNA, cyclin B1) at 1 ng/ml, and progesterone release at 1 and 10 ng/ml (all *p* < 0.05). In addition, LH enhanced the inhibitory effects of BTC on apoptotic markers (bax at 1–100 ng/ml; caspase-3 at 10 and 100 ng/ml), relative to the respective BTC dose (*p* < 0.05).

## Discussion

### BTC can affect ovarian granulosa cell functions

The ability of BTC to modulate proliferation and apoptosis in ovarian granulosa cells has been previously demonstrated in animal models [6, 7], and BTC has also been shown to stimulate prostaglandin synthase 2 (PTGS2) expression and PGE₂ production in human granulosa cells [8]. In the present study, BTC increased the levels of PCNA (a marker and regulator of the S-phase) and cyclin B1 (a marker of the G2-phase) [21], indicating that BTC can enhance ovarian cell proliferation across multiple cell-cycle stages. Additionally, BTC reduced the expression of bax and caspase-3 (markers of cytoplasmic apoptosis) [22], supporting an anti-apoptotic role for BTC. These findings support recent evidence highlighting the role of EGF-like ligands, including BTC, in regulating granulosa cell function and ovulatory processes [23]. The combined pro-proliferative and anti-apoptotic effects observed in human granulosa cells suggest that BTC may facilitate follicular growth and development by enhancing cell survival and division [24, 25]. In human granulosa-lutein cells, BTC upregulates pentraxin 3 (PTX3), a regulator of cumulus expansion during the periovulatory stage, via activation of EGFR and downstream ERK1/2 signaling, further supporting the functional relevance of BTC in human follicular processes [23]. Although the underlying mechanisms remain unclear, some effects of BTC may involve steroid hormones. In this study, BTC increased progesterone secretion, a hormone known to regulate proliferation, apoptosis, differentiation, and follicular and luteal function [24]. This suggests that BTC’s influence on granulosa cell behavior may be steroid-mediated. Enhanced steroid output may also reflect increased cell proliferation and viability induced by BTC.

Overall, our findings demonstrate that BTC exerts direct stimulatory effects on key ovarian functions—including proliferation, apoptosis regulation, and steroidogenesis—highlighting its potential relevance for human reproductive physiology. However, several limitations should be considered. The in vitro design of this study may not fully reflect the in vivo ovarian environment. In addition, species-specific differences should be taken into account, as BTC may exert variable effects across species. Therefore, further studies using i*n vivo* models and physiologically relevant conditions are needed to confirm the biological relevance of these findings.

### Gonadotropins can stimulate ovarian granulosa cell functions

Previous research has demonstrated that both FSH and LH enhance ovarian cell function by stimulating proliferation, steroid and peptide hormone production, folliculogenesis, and oogenesis, while reducing apoptosis [24, 26, 27]. Our findings in human ovarian cells are consistent with these observations: FSH and LH increased cell viability, promoted proliferation, elevated progesterone secretion, and suppressed apoptotic activity. These results reaffirm the essential roles of these gonadotropins in regulating key ovarian processes.

### Gonadotropins can promote the stimulatory action of BTC on ovarian granulosa cell functions

Gonadotropins not only affected key functional parameters but also modified BTC’s stimulatory effects on viability, PCNA, cyclin B1, bax, caspase-3, and progesterone release, indicating a functional interaction between these factors in the regulation of ovarian functions. BTC may act as a mediator or modulator of gonadotropin action. Although the precise intracellular mechanisms underlying this interaction remain unclear, it may involve key signaling pathways downstream of gonadotropin and growth factor receptors, particularly the ERK1/2 and PI3K/AKT pathways, which are known to regulate granulosa cell proliferation, survival, and steroidogenesis [28, 29, 30]. Analysis of these downstream signaling pathways would therefore be valuable in identifying potential molecular mediators linking BTC and gonadotropin action. Further studies are therefore warranted to investigate whether BTC and gonadotropins converge on these signaling cascades and to clarify the molecular basis of their combined effects.

Moreover, gonadotropins have been reported to transactivate the epidermal growth factor receptor (EGFR) pathway via induction of EGF-like ligands such as betacellulin, which subsequently activate EGFR signaling in granulosa and cumulus cells [14, 31, 32]. In addition, FSH has been shown to interact with EGFR signaling pathways, further supporting the existence of cross-talk between gonadotropin and EGFR signaling [33].

Therefore, we cannot exclude the possibility that the apparent combined effects of BTC and gonadotropins reflect convergence on a shared EGFR-mediated signaling pathway. The use of a specific EGFR inhibitor would be required to clarify this mechanism and would represent an important direction for future studies.

### Conclusions

Our findings demonstrate that BTC and gonadotropins (FSH and LH) can stimulate human ovarian cell function in vitro. In combined treatments, gonadotropins were able to modify some of the BTC-induced effects on cell viability, proliferation, apoptosis, and progesterone secretion. These results indicate a functional interaction between the actions of BTC and gonadotropins. However, further mechanistic studies are needed to clarify the underlying pathways involved in these interactions and to determine their physiological relevance i*n vivo*.

## Data Availability

The data supporting the findings of this study are available within the article.

## Abbreviations

BTC: Betacellulin
FSH: Follicle–stimulating hormone
LH: Luteinizing hormone
PCNA: Proliferating cell nuclear antigen
Bax: Bcl–2–associated X protein
ELISA: Enzyme–linked immunosorbent assay
DMEM: Dulbecco’s Modified Eagle Medium
PBS: Phosphate–buffered saline
HRP: Horseradish peroxidase
DAB: 3,3′–diaminobenzidine
IgG: Immunoglobulin G
CO₂: Carbon dioxide
PTGS2: Prostaglandin–endoperoxide synthase 2
PGE₂: Prostaglandin E2
EGFR: Epidermal growth factor receptor
ERK1/2: Extracellular signal–regulated kinase 1/2
PI3K: Phosphoinositide 3–kinase
AKT: Protein kinase B
PTX3: Pentraxin 3
